# Lemierre's Like Syndrome: Retropharyngeal Abscess With Internal Jugular and Cerebral Venous Thromboses and Septic Embolization Leading to Pulmonary Embolism and Cerebral Abscesses Complicated by Papilledema and Residual Sixth Cranial Nerve Palsy

**DOI:** 10.7759/cureus.56250

**Published:** 2024-03-16

**Authors:** Sathyaprakash Ranganath

**Affiliations:** 1 Emergency Medicine Department, University Hospital Wishaw, Wishaw, GBR

**Keywords:** lemierre's and lemierre's like syndrome, retropharyngeal abscess, sixth cranial nerve palsy, streptococcus intermedius, exotropia, cerebral abscess, septic embolism, cerebral venous thrombosis, jugular venous thrombosis, lalls

## Abstract

A male child with a history of sinusitis presented to the emergency medicine department with a high fever, neck swelling, headache, vomiting, and double vision. He was diagnosed with retropharyngeal abscess (RPA) with bilateral internal jugular vein (IJV) and cerebral venous thromboses. The child was treated promptly and transferred to a specialty center, where the abscess was drained. However, he developed papilledema and septic embolism, leading to pulmonary embolism and cerebral abscesses. The child was an inpatient for six weeks and had outpatient treatment for three months. He developed exotropia due to bilateral sixth cranial nerve palsy. This existed even at the 24-month follow-up. This case report highlights the rare complications and morbidity from the retropharyngeal abscess. It also emphasizes the early diagnosis and management options in a busy emergency medicine department.

## Introduction

Retropharyngeal abscess (RPA) is a rare but potentially fatal deep neck space infection. This is usually due to the suppuration of retropharyngeal lymph nodes with the spread of infection from the oral and upper respiratory tract in children, while in adults, it is usually due to the disruption of the oropharyngeal mucosa by a penetrating foreign body [1-4].

Lemierre's Syndrome (LS) combines pharyngeal infection, sepsis, and internal jugular vein (IJV) thrombosis caused by the obligate anaerobe *Fusobacterium necrophorum* and other *Fusobacterium* species. This is usually isolated from the drained pus or anaerobic blood cultures [5-7]. If other organisms are isolated (aerobic and other gram-negative), then the condition is described as Lemierre's Like Syndrome (LLS) [5].

The mortality and morbidity in RPA are due to the complications, which include airway obstruction, mediastinitis, jugular venous thrombosis, carotid artery stenosis or rupture, cervical osteomyelitis, spinal cord abscess, meningitis, and septic embolization [1-4,8,9]. Lemierre's and Lemierre's Like Syndrome (LALLS) are rare complications of oropharyngeal infections.

The presented patient developed both RPA and LLS along with other complications of septic embolization, which is very rare in itself.

## Case presentation

A 15-year-old male child presented to the emergency department (ED) complaining of high fever, left neck swelling, inability to open his mouth with neck pain, difficulty and pain when he opens his eyes, inability to walk straight, and double vision at times. Fourteen days before the ED arrival, he was treated for clinical features suggestive of sinusitis by his general/family practitioner.

A senior emergency physician evaluated the child with a high pediatric early warning score (PEWS) at triage. The child was ill-looking with a temperature of 40.5 degrees Celsius, tachycardia, ataxia, neck stiffness, photophobia, diplopia during right lateral gaze, and trismus. There was diffuse swelling on the left side of the neck and the postauricular area without any palpable lymphadenopathy. His Glasgow Coma Scale was 14/15, with eye-opening 3/4, verbal response 5/5, and motor response 6/6. His pupils were 3 mm and equally reacting to light. He weighed 65 kg.

The child was diagnosed with cerebral/meningeal irritation caused by an infection, but the ED physician could not explain why the child had trismus without tonsillitis or peritonsillar abscess. The child had a sore throat, and the provisional diagnosis was a "retropharyngeal abscess with infection extension to brain/meninges." After performing an initial assessment and establishing IV access, blood samples were collected for laboratory analysis, and blood cultures were sent. His venous blood gas was normal, with a lactate of 1.7 mmol/L. The hospital's sepsis protocol was followed, and the child was given IV antibiotics (benzylpenicillin 2.4 g, gentamicin 360 mg, and metronidazole 500 mg), IV fluids (0.9% saline), and oxygen. The child had significantly elevated inflammatory markers, with a white blood cell count of 22000 and a C-reactive protein (CRP) of 350.

**Figure 1:**
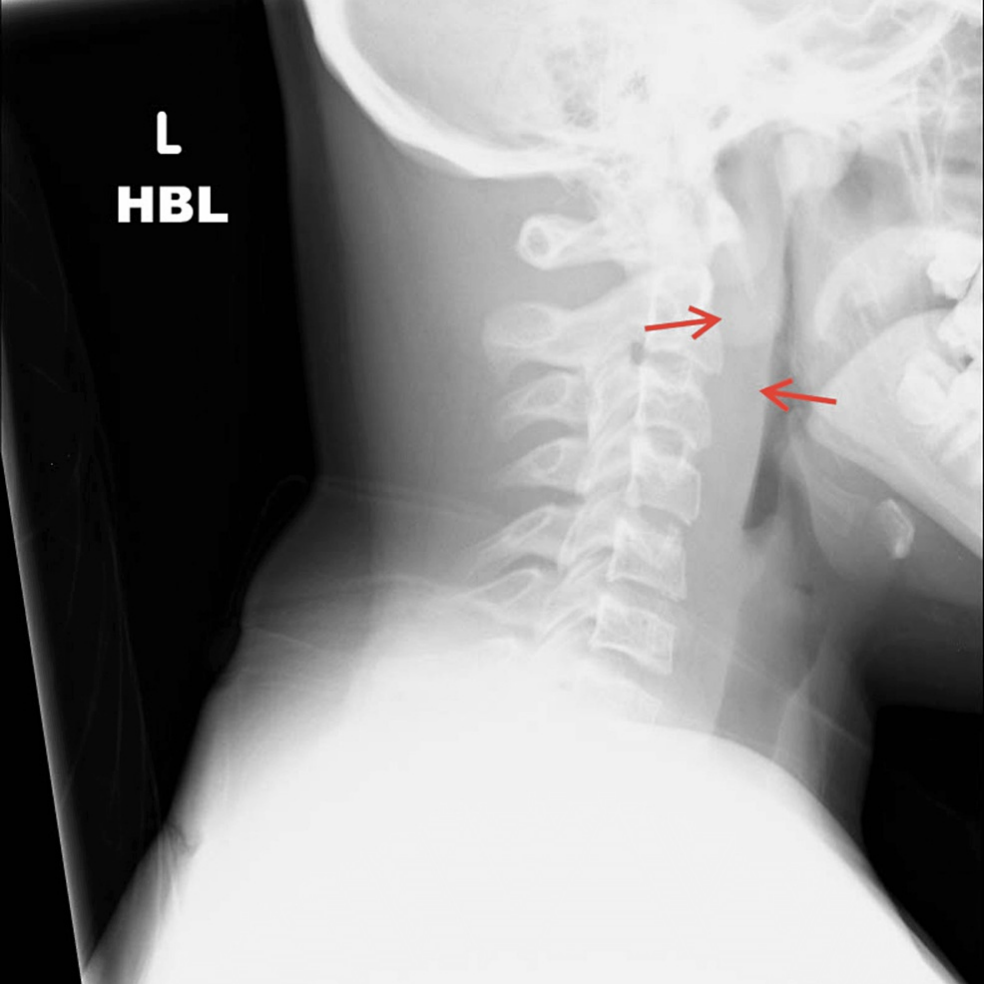
Lateral view of neck soft tissue exposure. The red arrows indicate the enlarged prevertebral space, which the radiologist reported as "nonspecific prominence of mostly cranial pre-cervical soft tissues." HBL: horizontal beam lateral

An X-ray of the lateral neck showed increased soft tissue swelling in the prevertebral areas of C1-4 (Figure 1).

**Figure 2:**
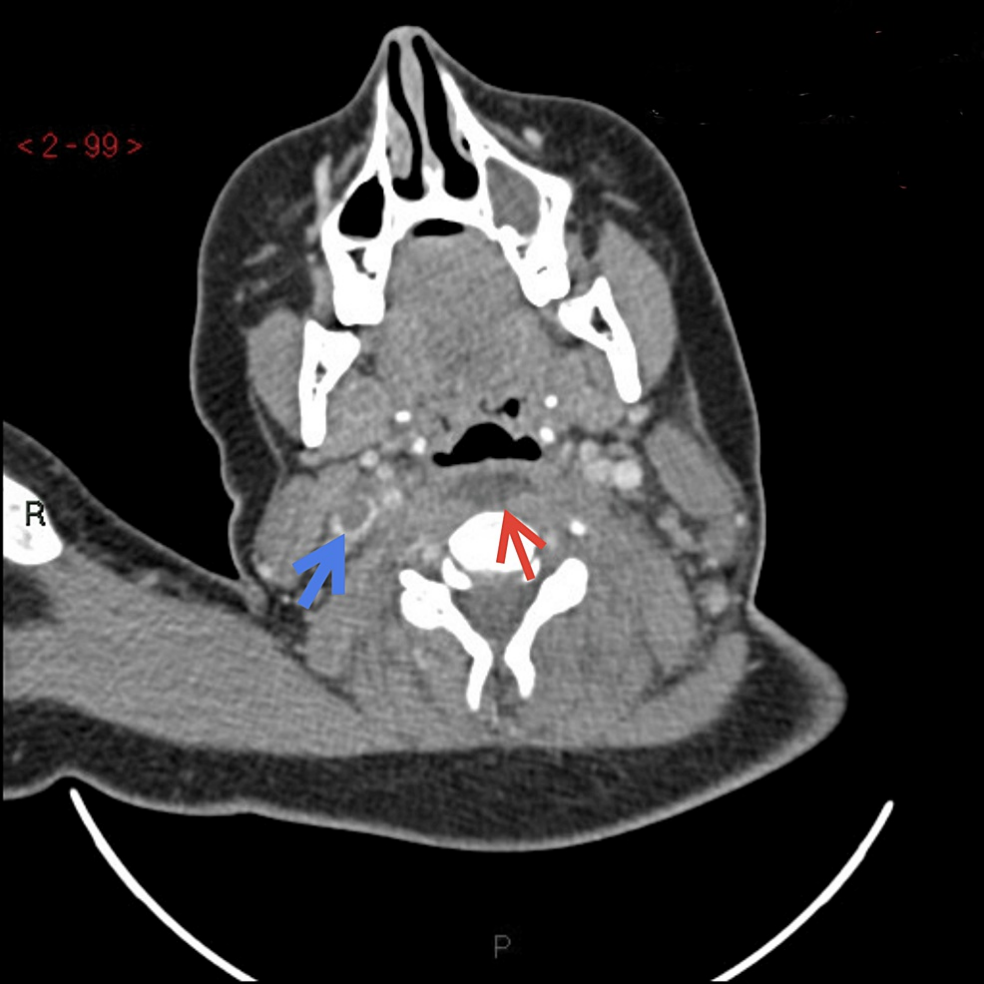
CT contrast study of neck transverse view. The blue arrow indicates the right internal jugular vein thrombus. The red arrow indicates retropharyngeal fluid suspicious of abscess formation.

**Figure 3:**
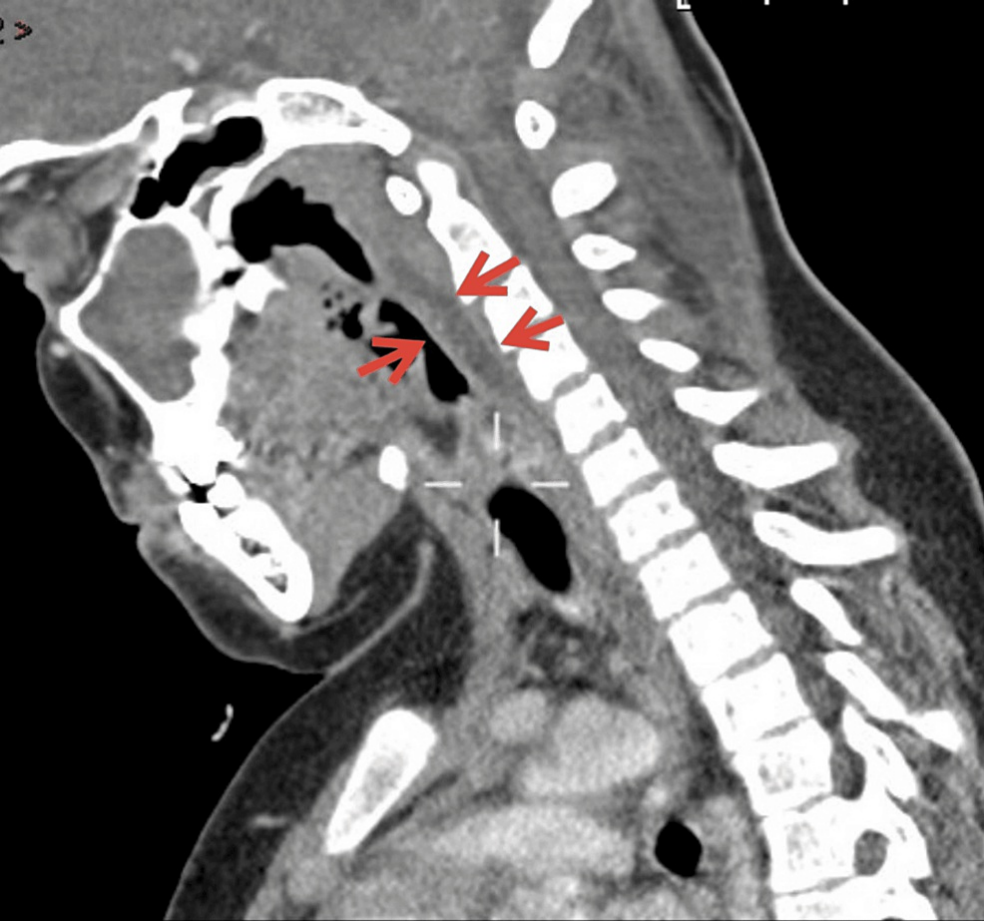
Contrast CT of the neck sagittal view. The red arrows indicate the presence of prevertebral or retropharyngeal collection.

**Figure 4:**
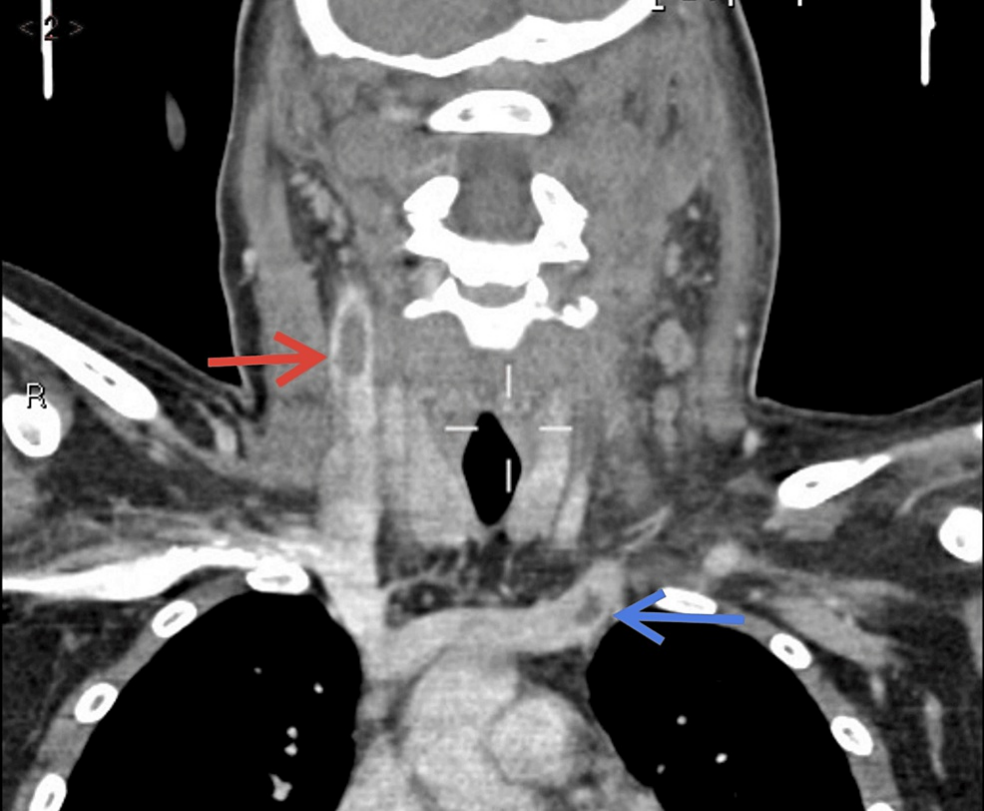
Contrast CT of the neck coronal view. The red arrow indicates a filling defect (thrombus) in the right internal jugular vein. The blue arrow indicates a filling defect (thrombus) at the junction of the left innominate and left internal jugular veins.

A contrast CT scan of the head/facial bones showed bilateral ethmoidal and left maxillary sinusitis with superior sagittal sinus thrombosis. A CT scan of the neck detected retropharyngeal fluid of size 3.5 cm×9 mm, suspicious for abscess formation (Figures 2, 3), and bilateral internal jugular vein thromboses, more extensive on the right (Figures 2, 4).

This child was then started on therapeutic low-molecular-weight heparin (LMWH) (enoxaparin 64 mg), and urgent ENT/otolaryngology and intensive treatment unit (ITU)/ICU/anesthetics referrals were done. He was intubated by the ITU team to protect the airway. He was transferred to a pediatric specialty hospital as he needed management from the pediatric ITU/ICU and ENT/otolaryngology teams with input from the hematologist and microbiologist.

In the specialty hospital, the ENT/otolaryngology team performed the drainage of the retropharyngeal abscess, the trephine of the left frontal sinus, and the middle meatal antrostomy of the left maxillary sinus. He was given IV fluids (crystalloids), IV antibiotics (ceftriaxone 2 g, clindamycin 670 mg, and metronidazole 500 mg), and therapeutic subcutaneous LMWH (enoxaparin 64 mg). The pus from the drainage and blood cultures grew gram-positive cocci after two days. By the fourth day, the type of cocci was identified as *Streptococcus intermedius*, which was found to be sensitive to penicillin, clindamycin, vancomycin, ampicillin/amoxicillin, and rifampicin.

On the fourth day of admission, he experienced chest pain and shortness of breath and required oxygen. A CT pulmonary angiogram confirmed a pulmonary embolus in the left lower lobe of his lung. Therapeutic anticoagulation was continued with LMWH.

**Figure 5:**
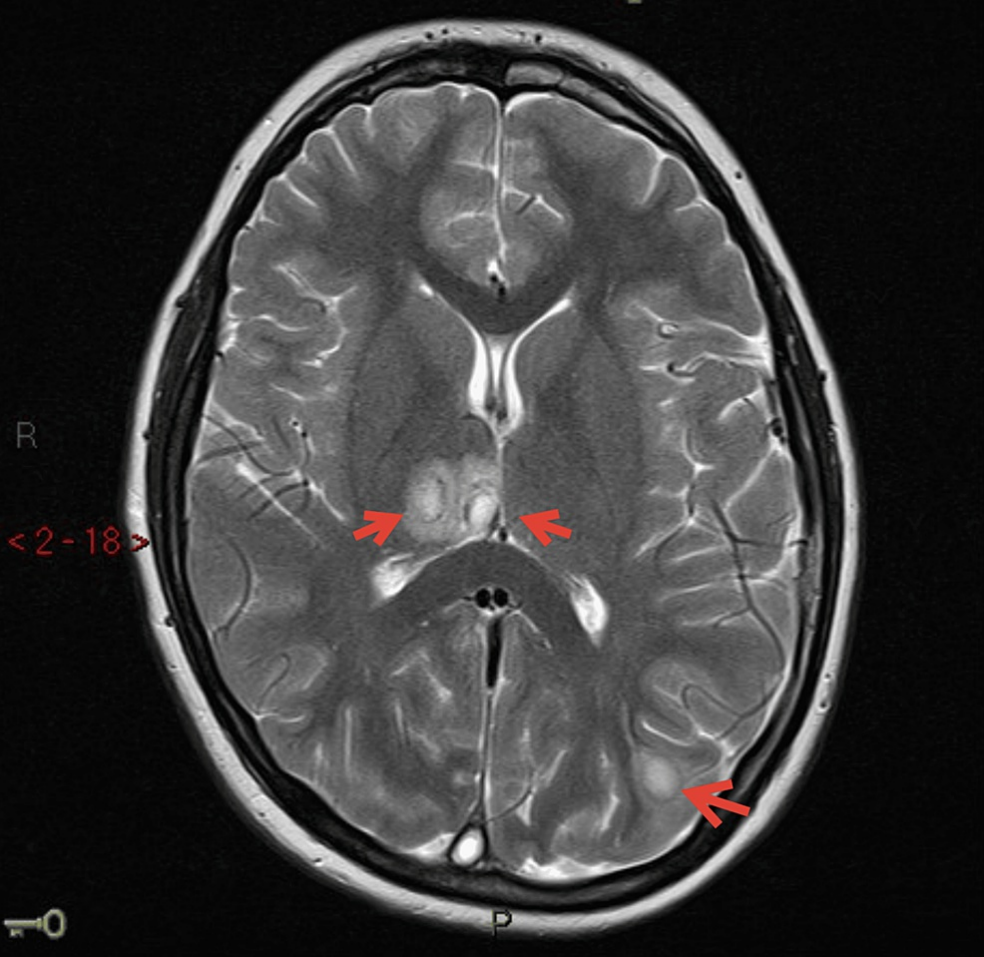
MRI of the brain with contrast. The red arrows indicate multiple ring-enhancing lesions in keeping with cerebral abscesses.

By the seventh day, he had worsening headaches, drowsiness, and double vision. He also had papilledema. An MRI of the brain was obtained, and the report indicated the presence of multiple ring-enhancing lesions in the left postcentral gyrus, both posterior parietal lobes, posteromedial right parietal lobe, and right thalamus. The largest lesion measured 11 mm, and these findings were consistent with cerebral abscesses (Figure 5).

The neurosurgeons took over the patient's care with a plan for conservative management. After consultation with the microbiologist, it was determined that the patient needed IV antibiotics for an extended period, and a peripherally inserted central catheter (PICC) line was installed for administering it.

After spending six weeks in the hospital, he was discharged when a repeated MRI scan showed a reduction of thrombus in his superior sagittal sinus and stable cerebral abscesses. During his hospital stay, he lost 10 kg of weight. District nurses visited him at home to administer IV antibiotics through the PICC line. For the next three months, he took IV ceftriaxone 4 g once daily, oral rifampicin 600 mg twice daily, and oral metronidazole 400 mg thrice a day. Additionally, he received enoxaparin 54 mg subcutaneously twice daily for six months.

Upon discharge from the pediatric specialty hospital, this patient received follow-up care from neurosurgeons, pediatricians, and ophthalmologists in their clinics for two years. The cerebral abscesses and internal jugular vein thromboses resolved within three and five months, respectively. However, at six months, he had residual papilledema and superior sagittal sinus thrombosis. At the 12-month follow-up, his papilledema had resolved, but he developed exotropia due to bilateral abducens nerve palsy, which persisted for up to 24 months. After 24 months, the patient did not attend any further follow-up appointments.

## Discussion

Clinical features

Lemierre's Syndrome was first identified by Andre-Alfred Lemierre, a French bacteriologist, in 1936. Its most common features include septic emboli (100%), thrombosis of the internal jugular vein (84%), sepsis (50%), a sore throat (24%), swelling in the neck (5%), and CNS affection (3.6%). However, this syndrome can be difficult to diagnose due to its low incidence and the absence of pathognomonic symptoms [5-7].

The common clinical features of a retropharyngeal abscess (RPA) are fever (90%-95%), cervical lymphadenopathy (76%-86%), neck swelling (38%-81%), torticollis (54%-67%), neck pain and reduced range of movement (45%-50%), preceding sore throat/upper respiratory tract infection (URTI) (33%-43%), odynophagia (43%), respiratory distress and drooling (10%-29%), and trismus (18%-24%) [2,8]. However, none of these features are specific to RPA. They can also be seen in other infections such as meningitis, epiglottitis, tonsillitis, peritonsillar abscess, and other neck infections/abscesses [1-4,8,9].

The patient who presented to our emergency department exhibited almost all the above symptoms, including photophobia, which was likely caused by meningeal irritation from the infection. After a few days, he began to experience other clinical features related to complications arising from septic embolization.

Diagnostic investigations

Diagnosing RPA and LALLS in the emergency department requires clinical expertise and high suspicion. It is important to confirm the diagnosis by conducting appropriate investigations. In the United Kingdom and Ireland, X-rays and CT scans are preferred as they are readily available, especially outside regular hours.

In the studies conducted by Al-Sabah et al. [9] and Page et al. [8], the CT scan sensitivity to detect RPA versus cellulitis was found to be 43% and 72%, respectively. In comparison, the specificity to detect RPA versus cellulitis was 63% and 59%, respectively. According to Page et al., out of 162 patients with RPA, 153 (94%) of them had a diagnostic CT scan, out of which 97 (63%) had an abscess, 26 (17%) had early abscess/phlegmon, and 30 (20%) had cellulitis in the retropharyngeal area [8]. Dawes et al. conducted a study on 21 RPA cases, in which a lateral X-ray of the neck was diagnostic in 12 (80%) cases while equivocal in three (20%) cases. In this study, 18 (95%) patients also had a diagnostic CT scan [2].

Based on the articles reviewed, it was found that out of the total 420 RPA patients, 409 (97%) were diagnosed through contrast CT scan, while only 12 (3%) had X-rays of the neck, and one (0.2%) had an ultrasound scan of the neck [2,3,8,9]. Out of the 260 patients with LALLS, 202 (78%) were diagnosed through contrast CT, while 37 (14%) had an ultrasound scan, and 15 (5%) had a contrast MRI scan [5-7].

**Figure 6:**
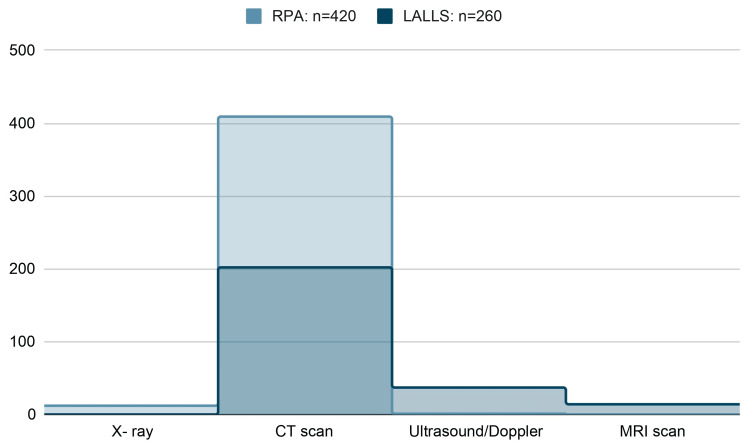
Radiological investigations obtained to diagnose RPA and LALLS. Image credits: Sathyaprakash Ranganath. RPA, retropharyngeal abscess; LALLS, Lemierre's and Lemierre's Like Syndrome

The above cohort's contrast CT scans proved to be the most appropriate radiological investigation for diagnosing RPA and LALLS (Figure 6).

In our patient, after obtaining the neck X-ray, we expedited the contrast CT scans as the image was not diagnostic of RPA. A CT scan of the brain/facial bones showed sinusitis and superior sagittal sinus thrombosis, while a CT of the neck confirmed the presence of a retropharyngeal collection and bilateral internal jugular vein thromboses. Thus, the diagnosis of RPA and the possibility of LALLS were ascertained. Obtaining the MRI or ultrasound scan with Doppler would not have made any difference in emergency department management. Moreover, it would have delayed the transfer to the specialty hospital. MRI scans of the head and neck were useful in diagnosing brain abscesses after a few days in the specialty hospital and later to monitor the resolution of these abscesses and multiple venous thromboses.

Management

While waiting for a definitive diagnosis and transfer to specialist care, it is crucial to provide adequate treatment for sepsis/infection and thrombosis and consider any potential airway compromise. It is recommended to consult with specialists in ITU, ENT, and infectious diseases to discuss further management options. If the patient requires care in a higher specialty hospital, the early consideration of safe transfer and the activation of a retrieval team, if available, are necessary.

Out of the 420 patients with RPA, 359 (85%) were treated with surgical drainage as the primary management approach while receiving antibiotics in the postoperative period. The remaining 61 (15%) were treated with antibiotics alone as the definitive treatment. However, among these patients, 17 (4%) required surgical drainage as their RPA progressed despite being treated with IV antibiotics [1-4,8,9].

All 260 patients diagnosed with LALLS were given antibiotics, as infections can increase the risk of thrombosis. In addition to antibiotics, 130 (50%) of LALLS patients received anticoagulation treatment in enoxaparin (LMWH) and/or warfarin. Anticoagulation aimed to maintain the anti-Xa activity between 0.5 and 1.2 U/dL and the international normalized ratio (INR) between 2.0 and 3.0. This treatment was continued for 3-6 months. Of the LALLS patients who received anticoagulation treatment, two (1%) developed hemorrhage and disseminated intravascular coagulation (DIC), while four (3%) experienced complete thrombosis resolution. However, three (2%) of the patients continued to have a persistent thrombus even after 12 months [5-7]. Johannesen and Bodtger reported no difference in death and the course of the disease between their anticoagulation (87/114, 76%) and no anticoagulation (27/114, 24%) treatment groups. However, they recommend aggressive anticoagulation treatment for patients with cerebral venous thrombosis than with jugular venous thrombosis [6]. Karkos et al. did not report any resolution or progression of thrombosis among their anticoagulation (41/137, 30%) and no anticoagulation (96/137, 70%) treatment groups [7]. The studies indicate that the risks and benefits of anticoagulation treatment in LALLS are not substantiated [5-7].

**Figure 7:**
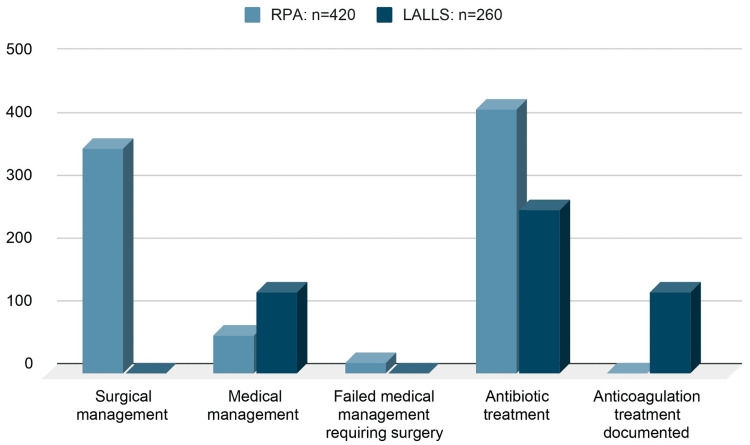
Treatment modalities chosen to manage RPA and LALLS. Image credits: Sathyaprakash Ranganath. RPA, retropharyngeal abscess; LALLS, Lemierre's and Lemierre's Like Syndrome

Figure 7 summarizes the treatment modalities chosen to manage the cases of RPA and LALLS in the above cohort.

After evaluation, our patient was administered IV benzylpenicillin, gentamicin, and metronidazole to cover gram-positive, gram-negative, and anaerobic organisms. After transfer to the pediatric ICU unit, he underwent surgical drainage of the RPA. In the postoperative period, he received IV ceftriaxone, IV clindamycin, and IV metronidazole for the first seven days. After seven days, clindamycin was discontinued, while IV ceftriaxone and metronidazole were continued. Due to the slow resolution of infections, especially the cerebral abscesses, the dosage of IV ceftriaxone was doubled, and rifampicin tablets were introduced to his antibiotic regimen in the third week. After being discharged from the hospital, he received IV ceftriaxone, oral metronidazole, and oral rifampicin for three months. By the end of that period, MRI scans showed the resolution of brain abscesses.

He was treated with LMWH enoxaparin twice daily by subcutaneous injection for six months to receive anticoagulation. His anti-Xa activity was maintained at 0.54 U/dL. By the end of the third month, most of the thromboses in the jugular veins had resolved. During the 12-month follow-up, he still had a nonocclusive thrombus in the superior sagittal sinus, which had improved from the previous MRI scan of six months.

Causative organisms

The appropriate antibiotic treatment for infection in the RPA and LALLS is determined based on the sensitivity and the microbial growth from the swabs taken from the throat, pus drainage, or blood.

Out of 420 patients with RPA, 368 culture and sensitivity samples were taken from throat and pus swabs. The growth of organisms was observed in these samples. Among the grown organisms, 197 (54%) were streptococci, out of which 105 (29%) were β-hemolytic, and 69 (19%) belonged to group A *Streptococcus*. The remaining 90 (24%) were other types of streptococci. Group B *Streptococcus* and group F *Streptococcus anginosus* species were each found in only one (0.3%) sample. The next most commonly isolated organism was *Staphylococcus aureus*, found in 54 (15%) samples. Of these, eight (2%) were methicillin-resistant *Staphylococcus aureus* (MRSA). Mixed anaerobes were found in 37 (10%) samples, and other organisms were isolated in 34 (9%) cultures. Other rare organisms that were isolated included *Neisseria* (23, 6%), *Haemophilus* (18, 5%), mixed growth (four, 1%), and atypical mycobacteria (one, 0.3%) [1-4,8,9].

Out of the 260 patients who had LALLS, swabs were taken for culture and sensitivity from 194 of them. Among these, 150 (77%) showed the growth of anaerobic *Fusobacterium* species, 21 (11%) had *Streptococcus* growth, 14 (7%) had mixed growth, and nine (5%) showed *Staphylococcus aureus* growth, which included six (3%) cases of MRSA [5-7].

**Figure 8:**
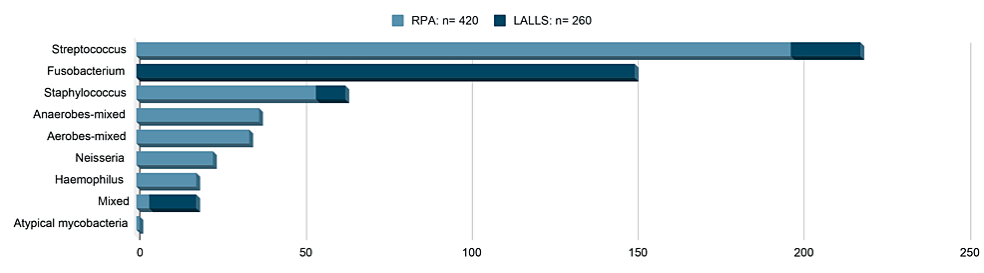
Organisms isolated from blood and swab cultures of RPA and LALLS pooled patients. Image credits: Sathyaprakash Ranganath. RPA, retropharyngeal abscess; LALLS, Lemierre's and Lemierre's Like Syndrome

In the RPA cohort, the most commonly identified organisms belong to the *Streptococcus* and *Staphylococcus* groups, while in the LALLS cohort, it was the *Fusobacterium* and *Streptococcus* groups (Figure 8).

The blood cultures of the presenting patient in the ED and pus cultures from drainage in the theatre revealed the growth of *Streptococcus intermedius*. The isolation of this bacterium in the presence of other clinical features confirms the diagnosis of Lemierre's Like Syndrome (LLS). *Streptococcus intermedius* is an aerotolerant facultative anaerobic commensal bacteria belonging to *Streptococcus anginosus* (group F). It is commonly isolated from patients with periodontitis and fatal purulent infections such as brain and liver abscesses [5-7]. This bacterium was sensitive to antibiotics such as penicillin, clindamycin, vancomycin, ampicillin/amoxicillin, and rifampicin. Our patient received the appropriate antibiotics at the ED, wards, and home.

Complications

Both RPA and LALLS patients are known to develop multiple complications.

Of 420 RPA patients, 59 (14%) morbidity events and no mortality were recorded. There was more than one event/complication in these patients. The most common complication was airway obstruction (30, 7%), followed by mediastinitis (11, 3%), and persistent abscess requiring repeat drainage (11, 3%). There were two (0.5%) nerve palsies (recurrent laryngeal and hypoglossal nerves) and one (0.2%) each of IJV thrombosis, IJV and internal carotid artery compression, vertebral osteomyelitis, and recurrent retropharyngeal cellulitis requiring further antibiotic therapy [1-4,8,9].

Among the 260 LALLS patients, there were eight (3%) mortalities, while morbidity was in all 260 (100%) patients. All 260 (100%) patients had septic embolization to other organs, leading to thrombus, infarction, abscesses, and infections. Septicemia was in 70 (26%) cases. Seventeen (7%) patients had to be intubated for airway obstruction, six (2%) patients had eye complications (uveitis, vitreous hemorrhage, retrobulbar mass, and sixth nerve palsy), and three (1%) patients had lower cranial nerve palsies involving 11th and 12th nerves [5-7].

**Figure 9:**
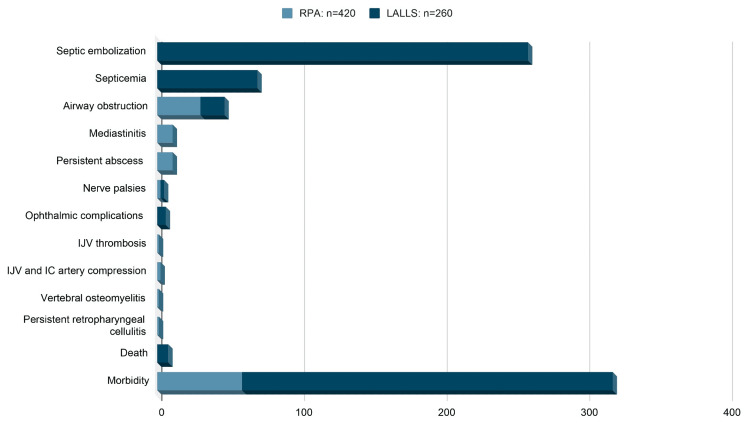
Mortality and morbidity among the RPA and LALLS cohort. Image credits: Sathyaprakash Ranganath. RPA*,* retropharyngeal abscess; LALLS, Lemierre's and Lemierre's Like Syndrome; IJV, internal jugular vein; IC, internal carotid

The above studies show that the LALLS group had higher morbidity and mortality compared to the RPA group. In the LALLS group, septic embolization and septicemia were common, while death was rare. Airway obstruction requiring intubation and cranial nerve palsies were common in both the RPA and LALLS patients (Figure 9).

The presenting patient had multiple morbidities and a protracted illness requiring prolonged treatment due to the presence of both RPA and LALLS. Apart from septicemia, he had multiple complications involving the brain (superior sagittal sinus thrombosis and abscesses), neck (RPA-deep neck abscess and venous thromboses), lungs (embolism), and eyes (papilledema and residual bilateral sixth nerve palsy leading to exotropia). Fortunately, his airway was not obstructed when he arrived in the ED. However, an airway was secured as he was ill and had to be transferred safely to the specialty hospital. Unfortunately, we do not know whether he still has exotropia or if he has developed any post-thrombotic syndrome (PTS).

## Conclusions

Diagnosing and managing retropharyngeal abscess (RPA) and Lemierre's and Lemierre's Like Syndrome (LALLS) are important in the emergency medicine department. To do this, take assertive action by being vigilant when treating a sick child and initiating sepsis management, including administering early antibiotics. Antibiotics coverage in the ED should include gram-positive, gram-negative, aerobe, and anaerobe organisms. Therapeutic anticoagulation treatment should be considered in the ED in the presence of central venous thrombosis with hematologist input for further management, considering the risks and benefits. Remember that a contrast CT scan is necessary to detect RPA and other pathologies. Ensure early specialty input to manage and secure the airway. Anticipate and treat complications early. Finally, ensure a safe transfer if needed.
